# Preparations from selected cucurbit vegetables as antiplatelet agents

**DOI:** 10.1038/s41598-021-02235-w

**Published:** 2021-11-22

**Authors:** Agata Rolnik, Bartosz Skalski, Anna Stochmal, Beata Olas

**Affiliations:** 1 Department of General Biochemistry, Faculty of Biology and Environmental Protection, University of Łódź, 90-236 Lodz, Poland; 2 Department of Biochemistry and Crop Quality, Institute of Soil Science and Plant Cultivation, State Research Institute, 24-100 Puławy, Poland

**Keywords:** Biochemistry, Cell biology

## Abstract

Increased blood platelet activation plays an important role in cardiovascular diseases (CVDs). Recent experiments indicate that certain fruits and vegetables, including onion, garlic, and beetroot, have anti-platelet potential and therefore may reduce the likelihood of CVDs. While vegetables from the *Cucuritaceae* family are known to exerting beneficial antioxidant and anti-inflammatory effects, their effects on blood platelet activation are poorly understood. Therefore, the aim of the present study was to determine the effect on platelet adhesion of preparations from selected cucurbits: pumpkin (*Cucurbita pepo*; fruit without seeds), zucchini (*Cucurbita pepo convar. giromontina*; fruit with seeds), cucumber (*Cucumis sativus*; fruit with seeds), white pattypan squash (*Cucurbita pepo var. patisoniana*; fruit without seeds) and yellow pattypan squash (*Cucurbita pepo var. patisoniana,* fruit without seeds). It also evaluates the activity of these preparations on enzymatic lipid peroxidation in thrombin-activated washed blood platelets by TBARS assay. The study also determines the anti-platelet properties of these five cucurbit preparations in whole blood by flow cytometry and with the total thrombus-formation analysis system (T-TAS) and evaluates the cytotoxicity of the tested preparations against platelets based on LDH activity. The results indicate that the yellow *Cucurbita pepo var. patisoniana* preparation demonstrated stronger anti-platelet properties than the other tested preparations, reducing the adhesion of thrombin-activated platelets to collagen/fibrinogen, and inhibiting arachidonic acid metabolism and GPIIb/IIIa expression on 10 µM ADP-activated platelets. None of the preparations was found to cause platelet lysis. Our findings provide new information on the anti-platelet activity of the tested cucurbit preparations and their potential for treating CVDs associated with platelet hyperactivity.

## Introduction

It is important to stop bleeding promptly after vessel injury, maintain blood in a fluid state, and eliminate blood clots when integrity is restored to the vascular system. This operation, known as *hemostasis*, is underpinned by a range of highly-conserved mechanisms in which blood plays a key role^1,2^. Under physiological conditions, vascular integrity is restored by clot formation at the injured site, and to prevent thrombosis, this process is balanced by various anticoagulant and antiplatelet mechanisms. An imbalance between the anticoagulant and procoagulant mechanisms may result in hemorrhage or excessive thrombosis. Thrombosis is a life-threatening occlusion of vessels, in which the normal feedback controls used to regulate thrombus size and stability no longer appear to function appropriately^3,4^. Blood platelets play a primary role in the first wave of hemostasis, also known as *primary hemostasis*; they also play an indirect one in the second wave, as mediators of the blood coagulation pathway. *Primary hemostasis* refers to the early stages of hemostasis when coagulation has to develop sufficiently to prevent blood loss during an injury. During this process, blood platelets interact with the exposed matrix, where they adhere to various adhesive proteins, including collagen^1,2^.

Blood platelets are the smallest cells in the circulation system, with a diameter of approximately 1–2 µm, and play a crucial role in hemostasis by repairing injuries to blood vessels. During their 8- to 12-day lifespan, they are mostly inactive^1^; however, they usually become activated after interacting with the protein receptors on the surface of the endothelium. This activation initiates a coagulation cascade: after adhesion to the extracellular matrix, von Willebrand factor from the exposed collagen forms a bridge with the platelet receptor complex glycoprotein (GP) Ib-IX-V. The collagen also binds to other platelet receptors, such as GPIa/IIa and GPVI leading to platelet activation and the release of P-selectin from α-granules in the platelets^5^.

The principal ligament for fibrinogen is the GPIIb/IIIa receptor (integrin α_IIb_β_3_), also known as CD41/CD61. It is a heterodimeric complex formed by the synthesis of a single IIb and single IIIs subunit. Beside fibrinogen, it can bind to fibronectin, von Willebrand factor, and vitronectin. The key function of integrin is to promote platelet aggregation by conducting bidirectional signals across the plasma membrane, made possible by its role in facilitating interactions with potential ligands. During its resting state, GPIIb/IIIa is bent and its “headpiece” is closed, thus reducing its affinity for physiological ligands; however, stimulation by certain stimulatory signals, such as from an inside-out signal, causes conformation changes that expose the extracellular binging domain^6,7^.

Platelet activation can be stimulated by multiple pathways; however, two key routes are connected with signal transduction pathways and have their foundation in membrane glycoproteins that are solely expressed in platelets. One pathway is based on the activation of platelets through G protein-coupled receptors, leading to the release of ADP and thromboxane A_2_, resulting in an increase of cytosolic calcium concentration. This initiates specific signaling pathways, and causes further platelet activation. Alternatively, the coagulation pathway generates thrombin, a highly potent platelet activator which is needed to convert fibrinogen into fibrin to stabilize the platelet “plug”. Thrombin activates blood platelets through protease activated receptors (PAR) on the platelet surface^1^. After activation, the platelets release P-selectin from α-granules to the surface. P-selectin is a ligand responsible for the interaction between platelets, leukocytes and endothelial cells, and plays a key role in linking hemostasis and inflammation^7^.

In addition, platelet activation can result in the activation of cytosolic phospholipase A_2_ (cPLA_2_), which generates arachidonic acid from phospholipid membranes. After formation, arachidonic acid is available for oxidization by cyclooxygenase (COX-1) or lipoxygenase (12-LOX). The results of oxidation are dependent on the type of blood cell. While oxidized arachidonic acid generates prostaglandin E_2_ and leukotriene B_4_ in leukocytes, it results in the production of thromboxane A_2_ and 12-hydroxy-5,8,10,14-eicosatetraenoic (12-HETE) in platelets^8^. Arachidonic acid can be also oxidized by cytochrome P 450, a heme-containing enzyme found in different tissues^9^. Hemostasis is not the only function of platelets, due to their high sensitivity to different diseases states, which make them one of the most accessible markers. Platelets interact with leukocytes and endothelium cells and their reactivity for various pathogenesis states are widely dependent upon active markers, including CD36, CD41, CD42a, CD42b, CD61. Platelets are also able to release and transfer many substances, which interact with endothelium cells. They can store high amount of amyloid precursor protein and its metabolism may accumulated Aβ in the brain, leading to its vasculature through blood brain barrier. In renal diseases platelets are in the presence of toxic products in the circulation leading to the form of bleeding diatheses, which are hemorrhage and other pathological feature like thrombocytopenia and glomerular thrombosis. Moreover, blood platelets play also an important role in metastasis^10^.

The members of the *Cucurbitaceae* family are rich in phenolic acids, flavonoids and terpenoids, all of which show strong antioxidant activity and may influence various parts of hemostasis. Oxidative stress is a risk factor in cardiovascular disease and hemostasis disorders. The components of cucurbits have been found to have a positive effect on biomarkers of oxidative stress in plasma^11^. Indeed, our previous studies have found selected vegetables from the *Cucurbitaceae* family, including pumpkin, zucchini, cucumber, yellow pattypan squash and white pattypan squash, to contain various secondary metabolites with antioxidant activity^11^; however, the influences of these vegetable preparations on the biological properties of blood platelets remain unknown. Therefore, to continue our previous research, the present study examined the anti-platelet potential of the same five cucurbit preparations, viz*.* pumpkin (*Cucirbita pepo*; fruit without seeds), zucchini (*Cucurbita pepo convar. giromontina*; fruit with seeds), cucumber (*Cucumis sativus*; fruit with seeds), white pattypan squash (*Cucurbita pepo var. patisoniana*; fruit without seeds) and yellow pattypan squash (*Cucurbita pepo var. patisoniana, fruit without seeds*) in tested washed human blood platelets and human whole blood in vitro. In addition to their anti-adhesive action, the present study also examines the activity of these preparations on enzymatic lipid peroxidation—arachidonic acid metabolism in thrombin-activated washed blood platelets based on thiobarbituric acid reactive substances (TBARS) assay. In addition, the present study also determines the anti-platelet properties of these five cucurbit preparations in whole blood using flow cytometry and a total thrombus-formation analysis system (T-TAS) and evaluates their cytotoxicity against platelets based on extracellular lactate dehydrogenase (LDH) activity. The action of cucurbit preparations was compared to commercial product—Aronox (*Aronia melanocarpa* berry extract with anti-platelet and antioxidant activities^12,13^.

## Materials and methods

### Chemical reagents

Dimethylsulfoxide (DMSO), bovine serum albumin (BSA), thiobarbituric acid (TBA), fibrinogen were acquired from Sigma-Aldrich (St. Louis, MO., USA). All other reagents were purchased from commercial suppliers, including POCH (Poland), Chempur (Poland), Chrono-log (Poland), and Kselmed (Poland). Ultrapure water was prepared in-house using a Milli-Q water purification system (Millipore, Milford, MA, USA).

A stock solution of commercial product—Aronox (*Aronia melanocarpa* berry extract, Agropharm Ltd., Poland) was prepared in H_2_O.

### Plant material

#### Obtained vegetable preparations

Five of the most well-known and easily-available types of cucurbit vegetables were selected for the study, these being pumpkin (*Cucurbita pepo *L., fruit without seeds); zucchini (*Cucurbita pepo *L.* convar. Giromontina*, fruit with seeds); cucumber (*Cucumis sativus* L., fruit with seeds); white pattypan squash (*Cucurbita pepo *L.* var. patisoniana*, fruit without seeds) and yellow pattypan squash (*Cucurbita pepo *L.* var. patisoniana*, fruit without seeds). All obtained materials were bought from organic farming in Poland 51°09′15.0″N 21°59′47.1″E, in 2019. The samples were shredded, frozen and freeze dried (CHRIST Gamma 2–16 LSC Freeze Dryer, Osterode am Harz, Germany), and stored in the Department of Biochemistry and Crop Quality of the Institute in Puławy, Poland. The plants were identified by Katarzyna Adamczyk: the owner of a private farm. The sample voucher for this material has been deposited in the Institute's collection under the deposit number 42/2019/IUNG, 43/2019/IUNG, 44/2019/IUNG, 45/2019/IUNG, and 46/2019/IUNG, respectively. All plant studies involved in the research were carried out in accordance with relevant institutional, national or international guideline. The entire section was previously described in Rolnik et al.^11^.

#### Extraction and chemical analysis of vegetable preparations

The extraction process was performed based on the following conditions: extraction solvent: 80% methanol, solvent pressure: 1500 psi, extraction cell temperature: 40 °C, extraction cycles: three, using an automatic extractor (Dionex ASE 200 Accelerated Solvent Extraction System). The extracts were dried by evaporation under reduced pressure, at 40 °C (HeidolphHei-Vap Advantage, rotary evaporator). The five preparations were purified from mostly sugars using solid phase extraction (SPE), as described previously^11^.

The most diverse phytochemical profile was demonstrated by the zucchini preparation, and the least by the cucumber. Almost all identified compounds could be classified as phenylethanoids, flavonoids, glycoside lipids or fatty acids. The pumpkin and cucumber contained, *inter alia*, kaempferol and synaptic acid; while the other three preparations only contained phenylethanoids as glycosides.

Both identified phenylethanoid glycosides, zizybeoside I and forsythoside E (isomer I), were present in zucchini. Of the identified phenolic acids, the sinapic acid hexoside was found in pumpkin and the salicylic acid O-glycoside in cucumber. Both pattypan squashes contain diterpenoids: cinncassiol A in the white pattypan and adenostemmoic acid C in the yellow pattypan. Among the flavonoids,7-methylquercetin-3-galactoside-6″-rhamnoside-3‴-rhamnoside, quercetin-3-O-rutinoside(rutin),isorhamnetin 3-O-rutinoside (narcissin) andhesperetin 7-O-(2″,6″-di-O-α-rhamnopyranosyl)-β-glucopyranoside, were identified.

Glycerophospholipids were identified in all cucurbit preparations. Fatty acids such as linoleic acid and octadecadienoic acid derivatives were also found in all the tested vegetables; however, the γ-linolenic acid derivative was present only in zucchini and yellow pattypan squash (Table 1)^11^.

**Table 1:** Identified compounds and their presence (+) in five cucurbit vegetable preparations based on Rolnik et al.^10^.

Identified compound	Compound class	Cucurbit preparation				
		Pumpkin	Cucumber	Zucchini	White pattypan squash	Yellow pattypansqaush
3-(β-d-glucopyranosyloxy)-2-hydroxybenzoic acid	Benzoicacidderivative	−	−	−	−	+
Fructosyl l-phenylalanine	Amino acid	+	+	−	+	+
l-tryptophan glycoside	Amino acid	+	−	+	−	−
Zizybeoside I	Phenylethanoid glycoside	−	−	+	−	−
Forsythoside E (isomer I)	Phenylethanoid glycoside	−	−	+	+	+
Hydrangeifolin I	Phenylpropanoid glycoside	−	−	−	+	+
Sinapic acid hexoside	Phenolic acid	+	−	+	−	−
Salicylic acid O-glycoside	Phenolic acid	−	+	−	−	−
Quercetin 3,3′-dimethyl ether 7-rutinoside	Flavonoids	−	−	+	−	−
Kaempferol derivative	Flavonoid	−	+	−	−	−
Primulaverin derivative	Flavonoid	−	−	+	−	−
Rutin	Flavonoid	−	−	+	−	−
Adenostemmoic acid C	Diterpenoids	−	−	−	−	+
Octadecadienoic acid derivative	Fatty acid	+	+	+	+	+
γ-Linolenic acid derivative	Fatty acid	−	−	+	−	+
Linoleic acid derivative	Fatty acid	+	+	+	+	+
Glycerophospholipid	Lipid	+	+	+	+	+

#### Stock solutions of vegetable preparation

To analyse the biological activity, stock solutions of the vegetable preparations were dissolved in 50% DMSO. The final concentration of DMSO in the samples (human plasma) was lower than 0.05% and its effects were determined in each experiment.

### Blood and blood platelets

#### Isolation of blood platelets

Human blood was collected from healthy, medication-free volunteers in the Medical Center in Lodz; all of whom reported not smoking or consuming alcohol. The blood was collected into tubes with citrate/phosphate/dextrose/adenine (CPDA) anticoagulant. Blood platelets were separated from fresh blood through differential centrifugation, as described previously^14,15^. Following this, the platelets were suspended in Barber’s buffer, in a modified Tyrode’s buffer (0.14 M NaCl, 0.014 M Tris, 10 mM glucose; pH 7.4). The amount of platelets used for the test reached 1.5–2.0 × 10^8^/mL and were measured using a UV–Visible Helios α spectrophotometer at 800 nm. For each experiment, blood or blood platelets were incubated for 30 min, at 37 °C with vegetable preparations at final concentrations of 5 and 50 μg/mL or aronia berry extract at final concentration of 50 μg/mL.

##### Confirmation by human participants

All experiments were approved by the University of Lodz Committee for Research on Human Subjects and carried out under permission number 8/KBBN-UŁ/III/2018.

We confirm that all experiments were performed in accordance with relevant guidelines and regulations. All donors were informed about the purpose of the study and gave their informed consent to participate.

### Effect of vegetable preparations on hemostasis parameters

#### Flow cytometry

To study the effects of the cucurbit preparations on the reactivity and activation of resting and stimulated blood platelets, whole blood models were used. Firstly, whole blood was incubated with preparations from selected cucurbit vegetables for 15 min at 37 °C, and then for another 15 min at room temperature (RT) with the addition of 10 and 20 μM ADP or collagen as platelet agonists. After incubation, the tested samples were diluted tenfold in sterile PBS with Mg^2+^, and then stained with 3 μL of anti-CD61/PerCP, anti-CD62/PE, or PAC-1/FITC antibodies for 30 min at RT in the dark. Isotype controls were also prepared; these contained resting blood samples stained with 3 μL of anti-CD61/PE and isotype control antibodies marked with FITC/PEisotype. Finally, all samples were fixed with 1% CellFix for 60 min at 37 °C.

The platelets were counted using an LSR II Flow Cytometer (Becton Dickinson, San Diego, CA, USA), based on the fluorescence of 10,000 platelets (CD61/PerCP positive objects). The platelets were distinguished from other blood cells by a forward light scatter (FCS) vs. side light scatter (SSC) plot on a log/log scale (first gate) and by positive staining with monoclonal anti-CD61/PerCP antibodies (second gate). The percentages of CD62P-positive and PAC-1-positive platelets were calculated in each sample. All results were analyzed using FlowJo _v.10.7.2 (Becton Dickinson, San Diego, CA, USA)^12,15,16,17^.

#### Total Thrombus Formation Analysis System (T-TAS)

T-TAS was used to determine the thrombus formation process under flow conditions using the PL-chip microchip coated with collagen. Fresh whole blood collected on BAPA (benzylsulfonyl-d-arginyl-prolyl-4-amidinobenzylamide) was incubated with preparations from the five cucurbit samples for 30 min at 37 °C. Then the samples were transferred to the PL-chip. The results were recorded as AUC_10_ i.e., Area Under the Curve^18^.

#### Platelet adhesion

Platelet adhesion was measured based on the activity of exoenzyme acid phosphatase in platelets. The plates were coated with 0.04 mg/mL collagen or 2 mg/mL fibrinogen. After isolation from fresh blood, the blood platelets were incubated with selected cucurbit preparations for 30 min at 37 °C. Next samples, which contain platelets and cucurbits preparations were added on plates and left to adhere for hour at 37 °C. The platelets were then dissolved with Triton X-100 and treated with the phosphatase substrate (p-nitrophenylphosphate), resulting in the formation of p-nitrophenol. The level of p-nitrophenol were measured at λ = 405 nm using a SPECTROstarNanoMicroplate Reader (96-well microtiter plates, BMG LABTECH, Germany). To achieve a color reaction in the samples, 2 M NaOH was added. All readings were taken in reference to the control sample containing only blood platelets with Barber’s buffer, in a modified Tyrode’s buffer, whose expression was assumed to be 100%^13,19^.

### Effect of vegetable preparations on parameters of damages

#### Activity of LDH

The cytotoxic effects of the selected *Cucurbitaceae* preparations on blood platelets were evaluated based on the release of lactate dehydrogenase (LDH) from the platelets. After incubation, the test samples were centrifuged for 15 min at 25 °C at 2500 rpm, and 10 μL of supernatant was transferred to a microtiter plate. The plate was then loaded with 270 μL of 0.1 M phosphate buffer and 10 μL of NADH. After a 20-min incubation at room temperature, 10 μL of pyruvate (5 mg) was added and the absorbance measured immediately afterwards. The further readings were taken at one-minute intervals over a 10-min period. Absorbance was measured at λ = 340 nm using a SPECTROstarNanoMicroplate Reader (BMG LABTECH, Germany)^20,21^.

### Effect of vegetable preparations on lipid peroxidation

The level of lipid peroxidation on the blood platelets was determined based on thiobarbituric acid reactive substances (TBARS) content. The samples were mixed with 0.37% thiobarbituric and 15% trichloroacetic acid and heated for 10 min at 100 °C in a heating block. Following this, the samples were allowed to cool and centrifuged at 10,000 rpm for 15 min at 18 °C. The absorbance of the supernatant was measured at λ = 535 nm using a SPECTROstarNanoMicroplate Reader (BMG LABTECH, Germany)^22,23^.

### Data analysis

Several tests were used to carry out the statistical analysis. All the values were expressed as mean ± SD. First the results were checked for normality with the Kolmogorow-Smirnow test, and the equality of variance was determined with Levine’s test. Statistically significant differences were identified using an ANOVA test (assuming a significance level of p < 0.05), followed by either Tukey’s multiple comparisons test or the Kruskal–Wallis test as appropriate.

## Results

### Effect of vegetable preparations on parameters of damages

**Figure 1:**
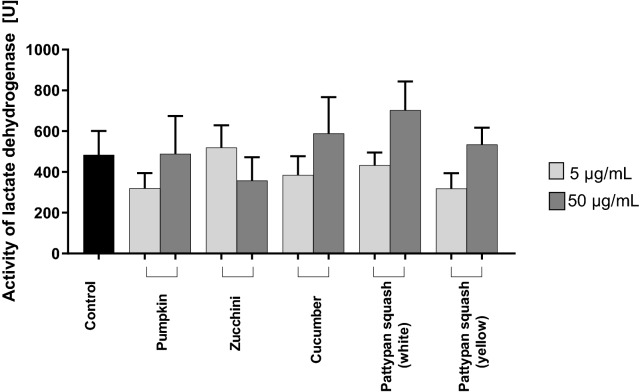
Effects of the five cucurbit vegetable preparations (concentrations 5 and 50 µg/mL, incubation time—30 min) on damage to human blood platelets. Results are given as mean ± SD (n = 4); the control sample (platelets without plant preparation). There wasn’t any statistically significant between effect of 5 and 50 µg/mL (p > 0.05). The baseline spectral reading (absorbance) for plant preparations range between 0.009 and 0.0245.

To determine the toxic effect of all the tested cucurbit preparations on human blood platelets, the level of extracellular LDH activity was measured. The results indicate no significant difference in blood platelet viability after exposure to the used plant preparations at 5 and 50 µg/mL compared to control, however, these changes were not statistically significant (Fig. 1).

### Effect of vegetable preparations on platelet adhesion

**Figure 2:**
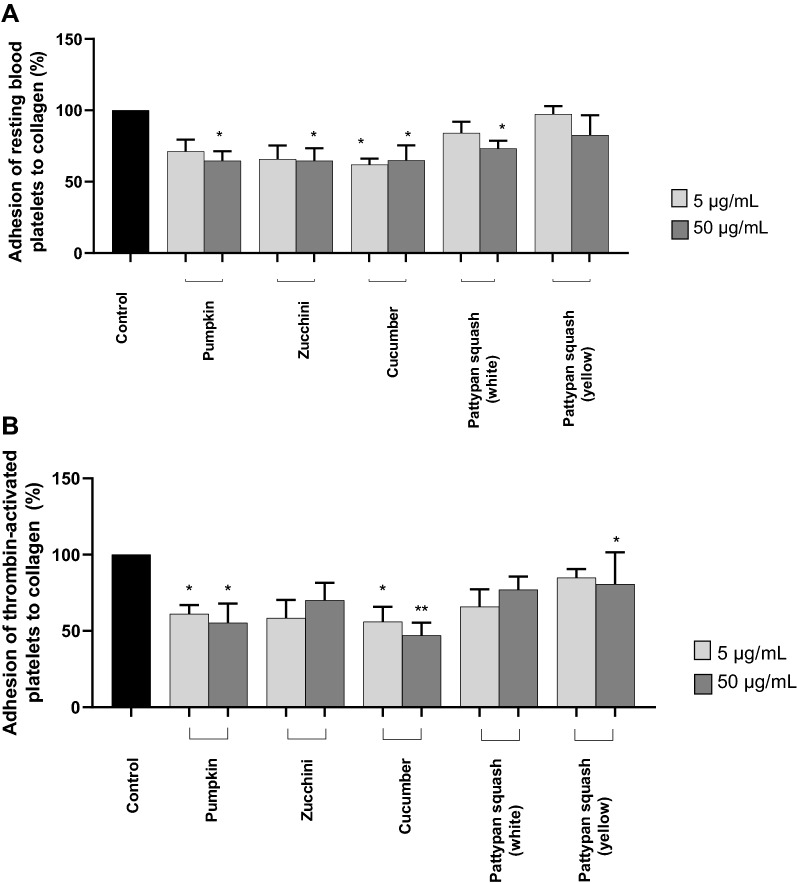
Effects of the five cucurbit vegetable preparations (concentrations 5 and 50 µg/mL, incubation time—30 min) on adhesion of resting platelets to collagen (**A**) or thrombin (final concentration 0.2 U/mL)—activated platelets (**B**). In the graphs, the adhesion is expressed as a percentage of the control sample (platelets without plant preparation). Results are given as mean ± SD (n = 5). Kruskal–Wallis test: *p < 0.05, **p < 0.01, compared with control (i.e. not treated with plant preparation). There wasn’t any statistically significant between effect of 5 and 50 µg/mL (p > 0.05). The baseline spectral reading (absorbance) for plant preparations range between 0.00075 and 0.0095.

The anti-adhesive properties of five plant preparations were studied in vitro using washed blood platelets. The results were presented as percent of level of adhesion for control samples. The obtained results showed the level of adhesion of resting platelets to collagen was significantly inhibited after pre-incubation with four tested preparations: pumpkin (50 µg/mL), zucchini (50 µg/mL), cucumber (5 and 50 µg/mL), and pattypan squash (white) (50 µg/mL) (Fig. 2A).

**Figure 3:**
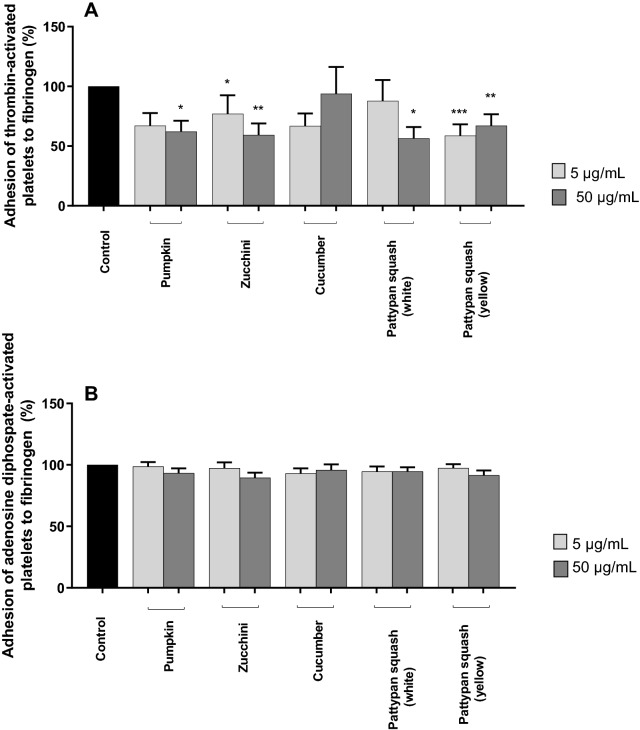
Effects of the five cucurbit vegetable preparations (concentrations 5 and 50 µg/mL, incubation time—30 min) on adhesion to fibrinogen and thrombin (final concentration 0.2 U/mL)—activated platelets (**A**) or ADP (final concentration 30 µM)—activated platelets (**B**). In the graphs, the adhesion is expressed as a percentage of the control sample (platelets without plant preparation). Results are given as mean ± SD (n = 5). Kruskal–Wallis test: *p < 0.05, **p < 0.01, ***p < 0.001, compared with control (i.e. not treated with plant preparation). There wasn’t any statistically significant between effect of 5 and 50 µg/mL. There wasn’t any statistically significant between effect of 5 and 50 µg/mL (p > 0.05). The baseline spectral reading (absorbance) for plant preparations range between 0.0025 and 0.0055.

In the case of the thrombin-activated blood platelets, both the pumpkin and cucumber preparations (5 and 50 µg/mL) also demonstrated inhibitory properties (Fig. 2B). Reduced adhesion was also observed for the pumpkin, cucumber, zucchini, pattypan squash (white), and pattypan squash (yellow) in other model (Fig. 3A); however, these changes were not always statistically significant, including cucumber (5 and 50 µg/mL), pumpkin (5 µg/mL), and pattypan squash (white) (5 µg/mL) (Fig. 3A).

For the ADP-stimulated platelets, all of the tested plant preparations (5 and 50 µg/mL) have not anti-adhesive properties (Fig. 3B).

### Effect of vegetable preparations on lipid peroxidation

**Figure 4:**
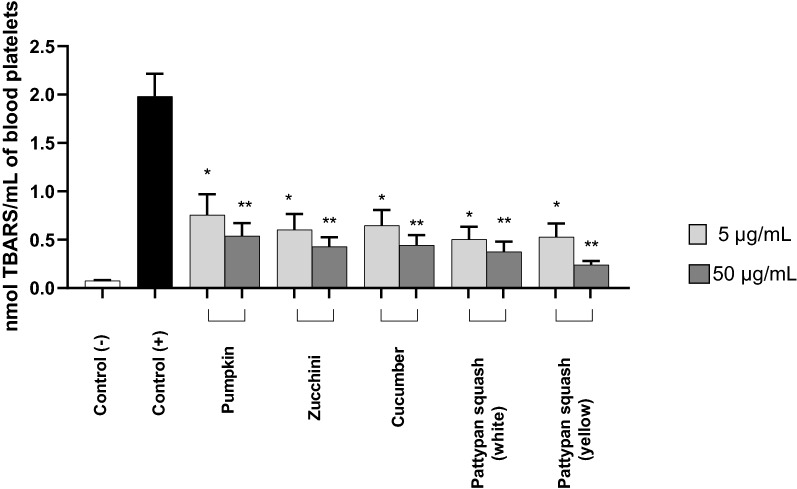
Effects of the five cucurbit vegetable preparations (concentrations 5 and 50 µg/mL) on lipid peroxidation in blood platelets activated by 5 U/mL thrombin (pre-incubation time with plant preparation—25 min; incubation time with thrombin—5 min). Results are given as mean ± SD (n = 5). *Control negative* refers to platelets not treated with thrombin, and *control positive* to platelets treated with thrombin. Kruskal–Wallis test: *p < 0.05, **p < 0.01. There wasn’t any statistically significant between effect of 5 and 50 µg/mL (p > 0.05). The baseline spectral reading (absorbance) for plant preparations range between 0.00065 and 0.0035.

As shown in Fig. 4, all the tested plant preparations (5 and 50 µg/mL) altered the level of lipid peroxidation in platelets stimulated by thrombin. Most significantly, the pattypan squash (yellow) preparation (50 µg/mL) demonstrated 85% inhibition relative to the positive control.

### Effect of vegetable preparations on the hemostasis parameters in whole blood

**Figure 5:**
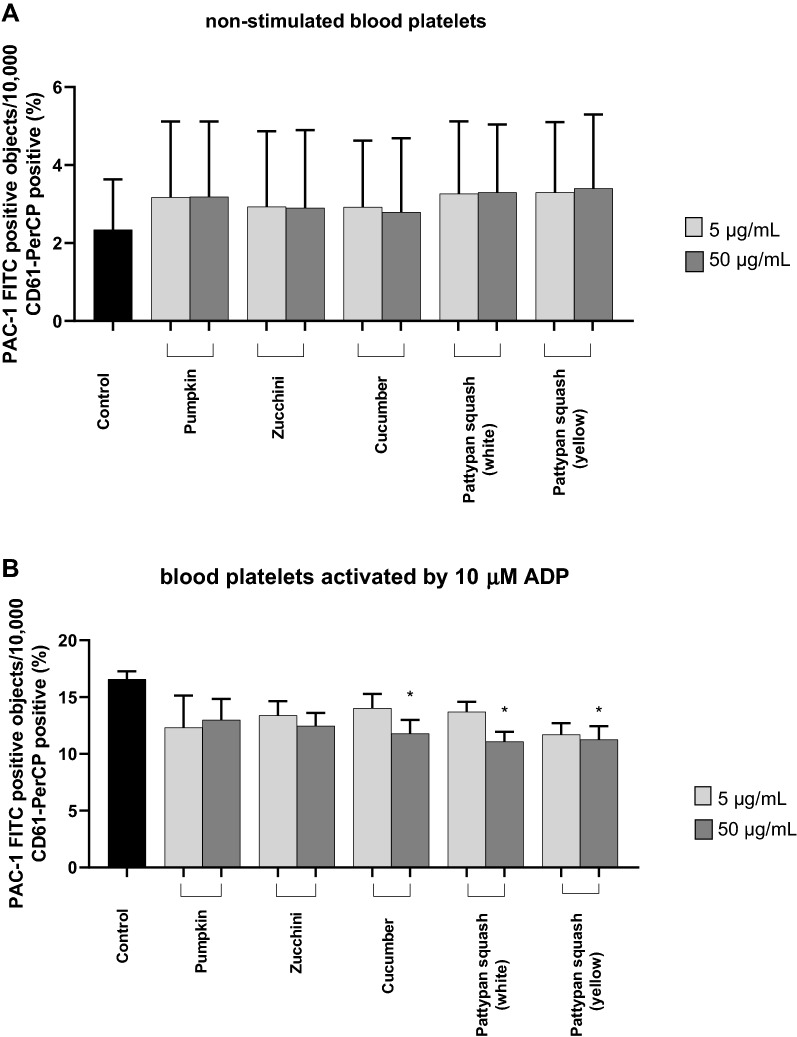

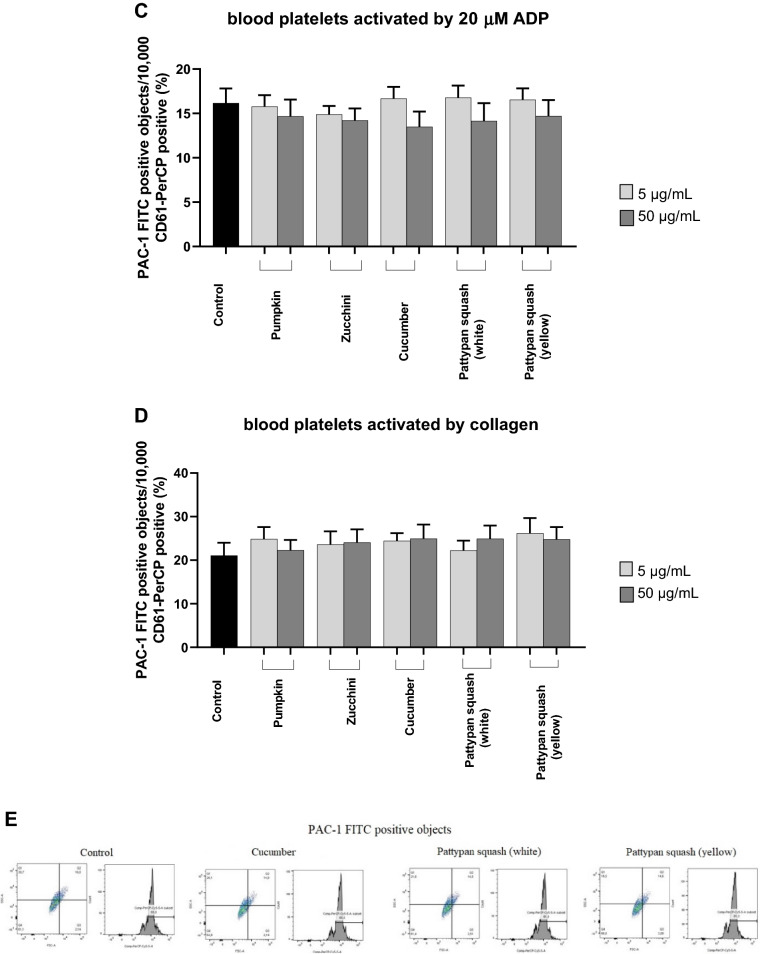
Effects of the five cucurbit vegetable preparations (concentrations 5 and 50 µg/mL, incubation time—30 min) on the expression of the active form of GPIIb/IIIa on resting (**A**) or agonist-stimulated blood platelets: 10 µM ADP (**B**), 20 µM ADP (**C**) and 10 µg/mL collagen (**D**) in whole blood samples. Additionally effects of three selected preparations (cucumber, pattypan squash white and yellow; 50 µg/mL; 30 min) on the expression of the active form of GPIIb/IIIa in platelets stimulated by 10 µM ADP in whole blood samples (**E**). This figure demonstrates selected diagrams (**E**). The blood platelets were distinguished based on the expression of CD61. For each sample, 10,000 CD61-positive objects (blood platelets) were acquired. For the assessment of GPIIb/IIIa expression, samples were labeled with fluorescently conjugated monoclonal antibody PAC-1/FITC. Results are shown as the percentage of platelets binding PAC-1/FITC. Data represent the mean ± SD of six healthy volunteers (each experiment performed in triplicate). *p < 0.05 (vs. control platelets). There wasn’t any statistically significant between effect of 5 and 50 µg/mL (p > 0.05).

**Figure 6:**
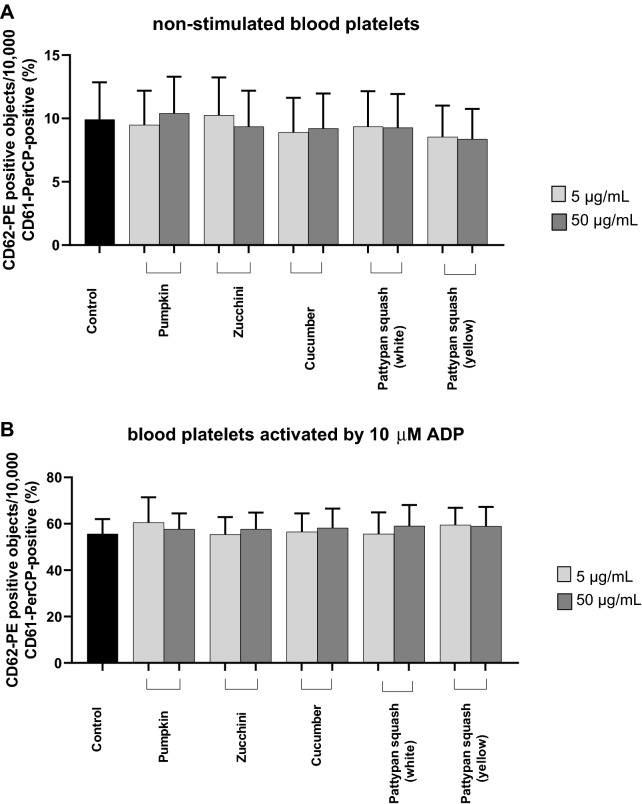

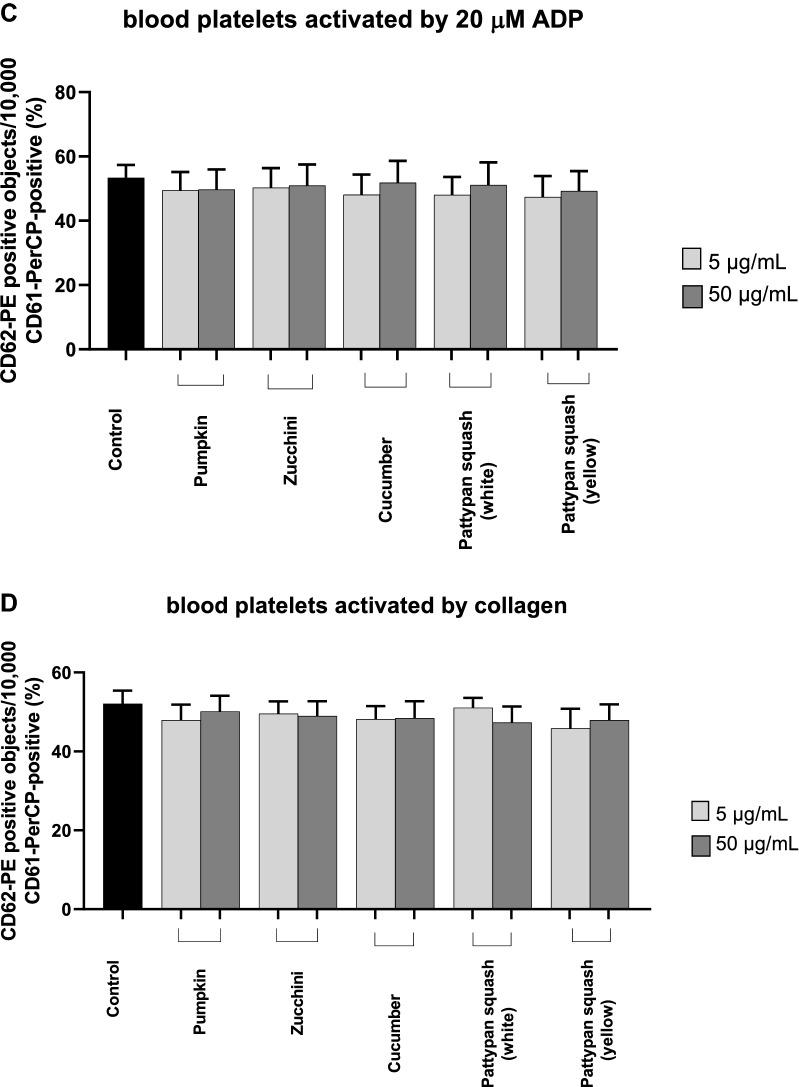
Effects of the five cucurbit vegetable preparations (concentrations 5 and 50 µg/mL, incubation time—30 min) on expression of P-selectin on resting (**A**) or agonist-stimulated blood platelets: 10 µM ADP (**B**), 20 µM ADP (**C**) and 10 µg/mL collagen (**D**) in whole blood samples. The blood platelets were distinguished based on the expression of CD61/PerCP. For each sample, 5000 CD61-positive objects (blood platelets) were acquired. For the assessment of P-selectin expression, samples were labeled with fluorescently conjugated monoclonal antibody CD62P. Results are shown as the percentage of platelets expressing CD62P. Results are given as the mean ± SD of six healthy volunteers (each experiment performed in triplicate). There wasn’t any statistically significant between effect of 5 and 50 µg/mL (p > 0.05).

The samples treated with five plant preparations demonstrated different levels of blood platelet activation (measured by flow cytometry) compared with untreated whole blood control samples (Figs. 5 and 6). Only three preparations demonstrated clear changes at the highest tested concentration (50 µg/mL): the cucumber, the pattypan squash (white), and pattypan squash (yellow) preparations significantly reduced PAC-1 binding in platelets activated by 10 µM ADP (Fig. 5B).

**Figure 7:**
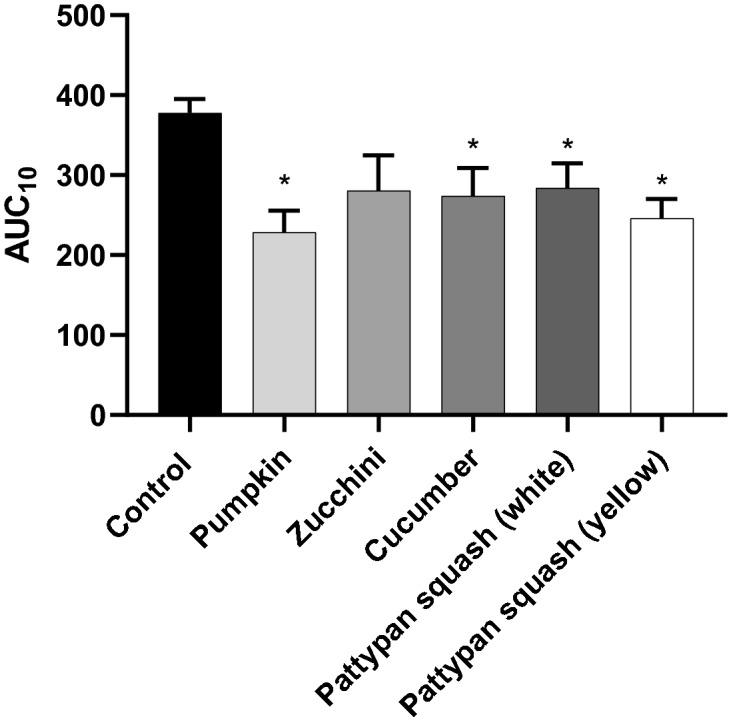
Effects of the five cucurbit vegetable preparations (concentration—50 µg/mL, incubation time—30 min) on the T-TAS using the PL-chip in whole blood samples. Whole blood samples were analyzed by the T-TAS at the shear rates of 1000 s^−1^ on the PL-chips. The area under the curve (AUC_10_) in PL are shown as closed circles. Data represent the mean ± SD of eight healthy volunteers (each experiment performed in triplicate). *p < 0.05 (vs. control blood).

Four cucurbit preparations (pumpkin, cucumber, pattypan squash (white), and pattypan squash (yellow)) significantly changed AUC_10_ values measured by T-TAS in whole blood (Fig. 7).

The effects of all five tested plant preparations (at the highest tested concentration) on chosen blood platelet activation parameters are compared with aronia berry extract in Table 2. Of all preparations, the yellow pattypan squash preparation demonstrated the strongest anti-platelet properties, reducing the adhesion of thrombin-activated platelets to collagen/fibrinogen, and inhibiting arachidonic acid metabolism and GPIIb/IIIa expression on 10 µM ADP-activated platelets. In addition, aronia berry extract (50 µg/mL) decreased PAC-1 binding in platelets activated by 20 µM ADP and collagen; it also reduced the expression of GPIIb/IIIa on platelets activated by 10 µM ADP, 20 µM ADP and collagen (data not presented).

**Table 2:** Comparation of influence of the five cucurbit preparations (tested concentration—50 µg/mL) on selected properties of activated blood platelets^11,12^.

Parameter of platelet activation	Cucurbit preparation					
	Pumpkin	Zucchini	Cucumber	Pattypan squash (white)	Pattypan squash (yellow)	Aronia berry extract
Adhesion of thrombin-activated platelet to collagen	Inhibition of this process (anti-platelet potential)	No effect	Inhibition of this process (anti-platelet potential)	No effect	Inhibition of this process (anti-platelet potential)	Inhibition of this process (anti-platelet potential)
Adhesion of thrombin-activated platelet to fibrinogen	Inhibition of this process (anti-platelet potential)	Inhibition of this process (anti-platelet potential)	No effect	Inhibition of this process (anti-platelet potential)	Inhibition of this process (anti-platelet potential)	No effect
Adhesion of ADP-activated platelet to fibrinogen	No effect	No effect	No effect	No effect	No effect	No effect
Arachidonic acid metabolism	Inhibition of this process (anti-platelet potential)	Inhibition of this process (anti-platelet potential)	Inhibition of this process (anti-platelet potential)	Inhibition of this process (anti-platelet potential)	Inhibition of this process (anti-platelet potential)	No effect
GPIIb/IIIa expression on 10 µM ADP-activated platelets	No effect	No effect	Inhibition of this process (anti-platelet potential)	Inhibition of this process (anti-platelet potential)	Inhibition of this process (anti-platelet potential)	No effect

## Discussion

A fundamental part of the primary and secondary treatment of atherosclerotic thrombotic disease is the use of drugs affecting platelet function. Although several such drugs are available in the clinical armory, aspirin and clopidogrel have the most favorable profile of currently-used drugs and are the most widely-studied. Aspirin plays a crucial role in preventing thromboembolic complications from atherosclerotic disease. It is believed to prevent platelet activation by permanently inactivating key platelet enzymes. In contrast, clopidogrel offers slightly better effectiveness regarding the secondary prevention of vascular events. While it has no direct antiplatelet activity of its own, it stimulates metabolites, like free thiols group to bind to P_2_Y_12_ platelet receptors to form disulfide bridges with extracellular cysteine residues, leading to irreversible inhibition of ADP-induced platelet aggregation, because the P2Y_1_ receptor plays an significant role in ADP -induced activation of platelets^24^.

In recent years the antiplatelet drugs are mostly related to role of mechanism of thrombus formation, which is exclusively expressed on blood platelet. Due to this important pharmacological research directions for treating hemostatic deficiencies are concerning the development of new drugs targeting, among others, the PAR1 thrombin receptor or GP VI platelet-specific collagen receptor^25^. The greatest adverse effect for antiplatelet drugs is the increased risk of bleeding, which is associated with an elevated risk of thrombosis. Antiplatelet therapy should inhibit platelet function during periods of high thrombotic risk. In addition, to avoid the risk of recurrence of ischemic events after premature cessation of medication or non-compliance, patients often require long-term antiplatelet therapy^24^.

The Cucurbitaceae family includes a range of phytochemicals, such as saponins and cardiac glycosides, which are known to influence on the cardiovascular system and are often used in the therapy of heart disease. Saponins are able to coagulate blood and reduce bleeding, and the cucurbitacins can exert an anti-atherosclerotic effect due to their ability to inhibit lipid peroxidation products, like malonaldehyde^26,27^. Cucurbitacin B has a protective effect against cardiac hypertrophy by increase autophagy among cardiomyocytes; while hypertrophy is a dynamic and adaptive process in physiological conditions, it can lead to pressure or volume overload, often resulting in heart failure if prolonged^28^.

The leaves and seeds of *Momoridace balsamina* represent a ready source of glycosides, which can be used to treat cardiac diseases by intensifying the force of heart contraction, based on its influence on calcium release. It has also been found that zucchini can also help alleviate symptoms related with heart diseases, such as high cholesterol^27^. Other research indicates that ethanolic extract of *Lagenaria siceraria* (*Cucurbitaceae*) fruit inhibits ADP-induced platelet aggregation in vitro, and increases bleeding time and plasma re-calcification time in mice^29^. Other experiments have found cucumber sap extract to inhibit blood platelet aggregation induced by different agonists (ADP, collagen, and epinephrine) in platelet-rich plasma and to decrease plasma re-calcification time and prothrombin time^30^. In addition, a recent study by Sanzana et al. found that various pumpkin seed extracts (aqueous, ethanolic and methanolic extract) have anti-platelet properties in vitro^31^; the extracts inhibited platelet aggregation stimulated by ADP, collagen and thrombin receptor activator peptide 6 (TRAP-6) in vitro, and reduced P-selectin expression and GPIIb/IIIa activation on blood platelets stimulated by TRAP-6^31^.

Our present findings, and those of our previous studies^11^, suggest that the selected cucurbit preparations offer promise as candidates for reducing blood platelet activation. It is worth noting that the present study used a combination of flow cytometry and T-TAS to study platelet activation in its natural environment, i.e. after blood collection and incubation with plant preparations, and that is the first paper to examine the anti-platelet potential of the five tested cucurbit preparations in two in vitro models: one based on washed blood platelets and the other with whole blood. An important, and novel, finding is that three tested cucurbit preparations: the yellow pattypan squash, white pattypan squash, and cucumber, influenced blood platelet activation, as indicted by flow cytometry. In addition, four cucurbit preparations (pumpkin, cucumber, pattypan squash (white), and pattypan squash (yellow)) changed AUC_10_ values measured by T-TAS. The differences in anti-platelet potential observed between samples may be explained by their different chemical profiles. An important consideration is that the concentration of plant preparation (≤ 50 µg/mL) used in the study corresponds with physiological concentrations of phenolic compounds after oral administration^32^.

Our results indicate that the yellow pattypan squash preparation appears to demonstrate the best anti-platelet properties of the tested cucurbits. The preparation was found to inhibit adhesion of thrombin-activated platelets to collagen and fibrinogen. It also significantly inhibited GPIIb/GPIIIa activation in ADP-activated platelets; it is likely that this inhibition may be responsible for the anti-adhesive potential of this preparation. This plant preparation was found to demonstrate anti-platelet potential measured by T-TAS. Again, its strong antiplatelet activity may well be correlated with its chemical composition. Preparations from pattypan squash are include a range of phenolic derivates, many of which show anti-platelet activity. One of the main compounds found in pattypan squash was phenylpropanoid glycoside, known to demonstrate anti-platelet activity^33^. Mesa et al. report that *Wendtia calycina* extract significantly reduced platelet aggregation induced by collagen in platelet-rich plasma in vitro. This extract contains a high level of phenylpropanoid glycoside, and a certain amount of benzoic acid derivates^33^, both of which were identified in pattypan squash (Table 1), with the benzoic acid derivative in the pattypan squash demonstrating a strong anti-aggregation effect^33^. The batch variability of plant matter is connected with the quantitative differences in chemical composition, due to the various plant cultivation methods and soil quality.

Pattypan squash also contains various diterpenoids, which are also known to demonstrate anti-platelet activity (Table 1). For example, Thisoda et al.^34^ found diterpenoids from *Andrographi spaniculate* extract to demonstrate anti-aggregatory effects of in vitro and to significantly inhibit thrombin-induced platelet aggegation^34^. However, further studies are needed to precisely identify the compound responsible for anti-platelet activity in pattypan squash. It may well be the case that this effect is derived from the synergistic action of multiple compounds together. Nevertheless, our results provide new information on the anti-platelet activity of the tested cucurbit preparations, especially the yellow pattypan squash preparation, and their possible use in CVDs associated with platelet hyperactivity.
